# Novel *ENAM* and *LAMB3* Mutations in Chinese Families with Hypoplastic Amelogenesis Imperfecta

**DOI:** 10.1371/journal.pone.0116514

**Published:** 2015-03-13

**Authors:** Xin Wang, Yuming Zhao, Yuan Yang, Man Qin

**Affiliations:** Department of Pediatric Dentistry, Peking University School and Hospital of Stomatology, Beijing, China; Huashan Hospital, Fudan University, CHINA

## Abstract

Amelogenesis imperfecta is a group of inherited diseases affecting the quality and quantity of dental enamel. To date, mutations in more than ten genes have been associated with non-syndromic amelogenesis imperfecta (AI). Among these, *ENAM* and *LAMB3* mutations are known to be parts of the etiology of hypoplastic AI in human cases. When both alleles of *LAMB3* are defective, it could cause junctional epidermolysis bullosa (JEB), while with only one mutant allele in the C-terminus of LAMB3, it could result in severe hypoplastic AI without skin fragility. We enrolled three Chinese families with hypoplastic autosomal-dominant AI. Despite the diagnosis falling into the same type, the characteristics of their enamel hypoplasia were different. Screening of *ENAM* and *LAMB3* genes was performed by direct sequencing of genomic DNA from blood samples. Disease-causing mutations were identified and perfectly segregated with the enamel defects in three families: a 19-bp insertion mutation in the exon 7 of *ENAM* (c.406_407insTCAAAAAAGCCGACCACAA, p.K136Ifs*16) in Family 1, a single-base deletion mutation in the exon 5 of *ENAM* (c. 139delA, p. M47Cfs*11) in Family 2, and a *LAMB3* nonsense mutation in the last exon (c.3466C>T, p.Q1156X) in Family 3. Our results suggest that heterozygous mutations in *ENAM* and *LAMB3* genes can cause hypoplastic AI with markedly different phenotypes in Chinese patients. And these findings extend the mutation spectrum of both genes and can be used for mutation screening of AI in the Chinese population.

## Introduction

Amelogenesis imperfecta (AI) is a group of inherited diseases that exhibit enamel malformations with diverse phenotypes and genetic heterogeneity [1–3]. The term is also applied to indicate the presence of an enamel phenotype in syndromes [4]. AI has been categorized as hypoplastic, hypocalcified, hypomaturized, and hypoplastic-hypomaturized types [1]. To date, mutations in the *AMELX*, *ENAM*, *AMBN*, *MMP20*, *KLK-4*, *FAM83H*, *WDR72*, *SLC24A4*, *C4orf26*, *ITGB6*, and *LAMB3* genes have been found to cause non-syndromic AI in human patients [5–14]. Among these, *ENAM* (OMIM 606585) and *LAMB3* (OMIM 150310) mutations can both result in hypoplastic AI and be inherited in an autosomal dominant mode, though their roles in the process of enamel formation may be quite different.

The *ENAM* gene encodes the largest (∼200 kDa) and also the least abundant (3–5%) enamel protein among three major extracellular matrix proteins in developing tooth enamel [15]. Previous reports supported the hypothesis that enamel defects caused by *ENAM* mutations are dose-dependent and result in hypoplastic AI, characterized by regions of abnormally thin or even absent enamel when both alleles are defective [16].

*LAMB3* encodes the beta-3 subunit of laminin, which belongs to a family of basement-membrane proteins. Mutations in both *LAMB3* alleles have been reported as the cause of various types of epidermolysis bullosa [17]. Since a frame-shift mutation in a single *LAMB3* allele was revealed to be the etiology of non-syndromic AI, a total of 5 mutations in *LAMB3* have so far been identified in different populations with an autosomal-dominant inheritance pattern[13,18,19].

To date, few studies have investigated the causative mutations of AI in Chinese populations. Here, we report three novel *ENAM* and *LAMB3* mutations (two *ENAM* and one *LAMB3*) causing hypoplastic autosomal-dominant AI in Chinese patients. This is the first time that a LAMB3 mutation has been identified in Chinese individuals with non-syndromic AI.

## Materials and Methods

### Study Participants

Three unrelated Chinese families segregating hypoplastic AI, but without any systemic diseases were recruited for genetic studies. All affected participants denied a history of living in an area with high fluoride in drinking water before age 8. No premature birth or problems during pregnancy were reported from each proband's mother. Clinical and radiographic examinations were performed and peripheral blood samples (4ml for each participant) were collected with the understanding and written consent of all participants including the guardians on behalf of the minors enrolled according to the Declaration of Helsinki. The protocol was reviewed and approved by the Ethics Committee of Peking University School and Hospital of Stomatology (PKUSSIRB-201311083).

### DNA extraction

Genomic DNA was extracted from peripheral whole blood using of a TIANamp Blood DNA mini kit (Tiangen, Beijing, China) according to the manufacturer's instructions.

### Mutation analysis

The entire coding region and adjacent intron boundaries of the *ENAM* and *LAMB3* genes were amplified by polymerase chain reaction (PCR) with TaKaRa Ex-Taq (Takara Bio, Kyoto, Japan). Primers were designed with Primer 3 on the Web (http://bioinfo.ut.ee/primer3-0.4.0/) (see S1 and S2 Tables). The products were purified and sequenced using an ABI 377 Automatic Sequencer (Applied Biosystems, Foster City, CA, USA). Sequencing was performed in both directions to confirm the mutation when an alteration was detected. We analyzed the insertion/deletion mutation with the help of Mutation Surveyor(SoftGenetics, State College, PA, USA).

### Protein structure analysis

The structures of the wild-type and mutant LAMB3 protein were modeled on the I-TASSER sever[20] and analyzed by CCP4MG [21] (S1 Fig.). Molecular graphics were created using PyMOL (The PyMOL Molecular Graphics System, DeLano Scientific, Palo Alto, CA, USA; http://www.pymol.org).

## Results

### Clinical findings

#### Family 1

**Fig 1:**
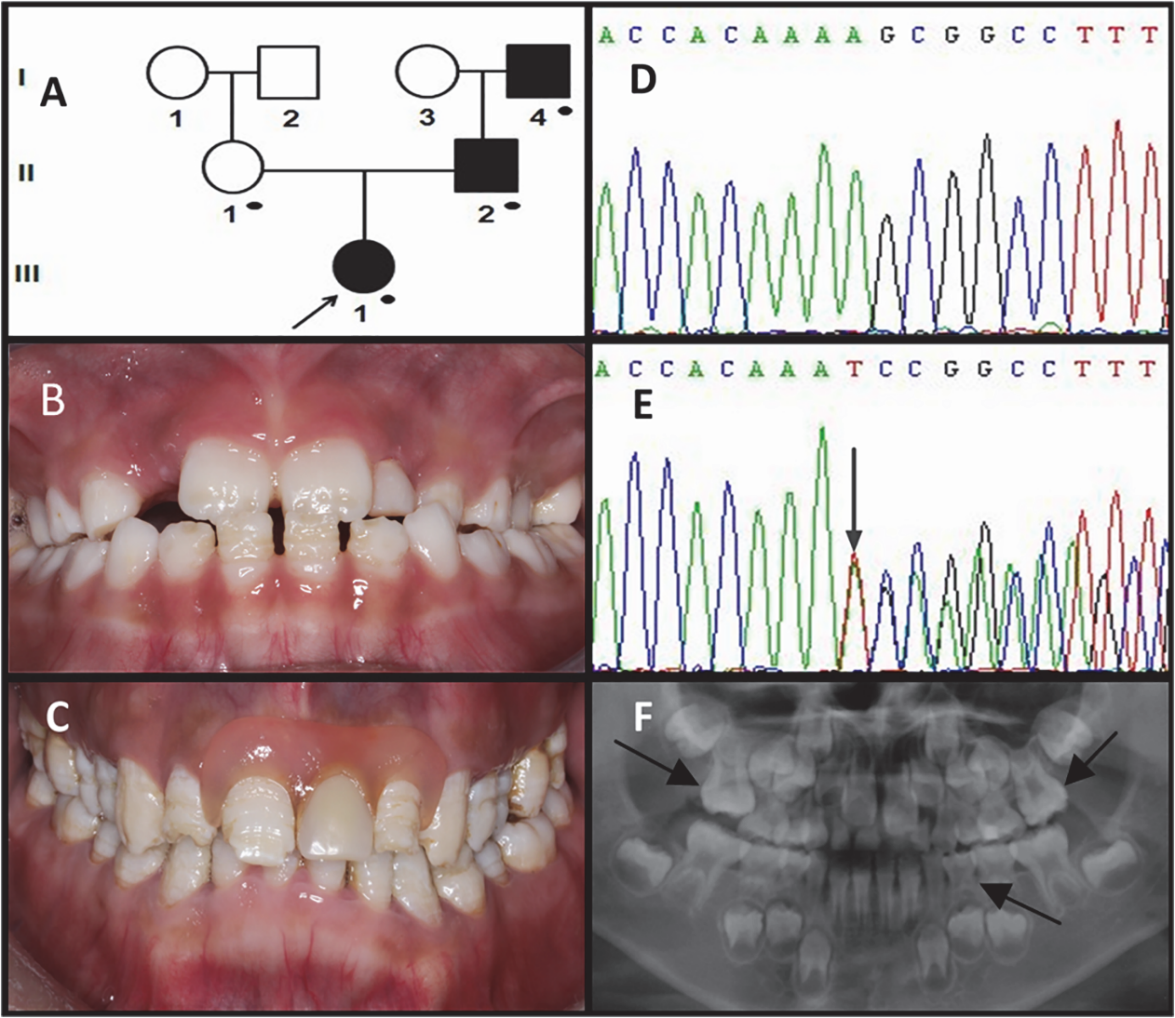
Clinical and mutation analysis of Family 1. (**A**) Pedigree of Family 1. Black dots indicate members recruited for this study. (**B**) Frontal clinical photograph of the 7.5-year-old proband. (**C**) Frontal clinical photograph of the proband's father. (**D, E**) *ENAM* exon 7 sequencing chromatogram of an unaffected family member (II:1) (D), and the proband (III:1) (E), revealed a 19-bp insertion mutation: c.406_407insTCAAAAAAGCCGACCACAA, p.K136IfsX*16. (**F**) Panoramic radiograph of the proband taken at the age of 6.5.

The clinical phenotype of this family was typical hypoplastic pitting and horizontal grooves. The proband was a 7.5-year-old girl presenting with grooved and pitted hypoplastic enamel in her newly-erupted permanent incisors without her primary teeth being affected. The spacing of the mandibular anterior teeth was clearly secondary to the thin enamel on the crowns. Anterior crossbite was noted though the maxillary incisors have not completely erupted (Fig. 1B). Radiographic examination showed generally thin enamel especially in anterior teeth and unerupted canines. The radiographic density of the enamel was normal or similar in density to the underlying dentin. Signs of taurodontism in the first permanent molars as well as in the mandibular first primary molars could also be seen (Fig. 1F). The enamel of the proband's father showed typical horizontal grooves on the buccal and lingual/palatal surfaces (the left maxillary incisor was a removable denture). Chips were visible on the incisal edges of the maxillary incisors. No occlusal problems were found in this participant (Fig. 1C). The proband’s grandfather (I-4) had worn a maxillary and a mandibular complete denture for at least 5 years (data not available). He reported that he had dental problems similar to those of his son, indicating a dominant pattern of inheritance.

#### Family 2

**Fig 2:**
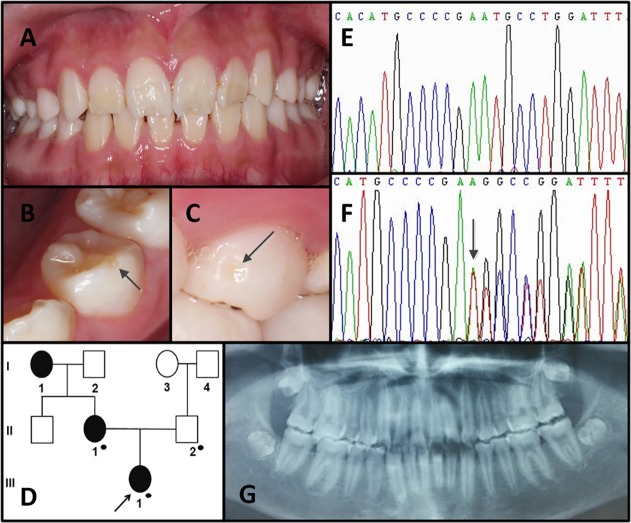
Clinical and mutation analysis of Family 2. (**A**) Frontal clinical photograph of the 13-year-old proband. (**B, C**) Representative examples of enamel pitting in the proband are illustrated by the arrows in the photographs. (**D**) Pedigree of Family 2. Black dots indicate members recruited for this study. (**E,F**) *ENAM* exon 5 sequencing chromatogram of the proband's father (II-2) (E), and the proband (III-1) (F), revealed a single-base deletion mutation: c. 139delA, p. M47Cfs*11. (**G**) Panoramic radiograph of the proband.

The proband was a 13-year-old girl who presented to our department due to the sensitivity to cold and hot stimuli. Her enamel defect was localized hypoplasia mainly involved the incisors and was particularly thin on the incisal 1/2 of maxillary anterior teeth (Fig. 2A). Her other teeth seemed normal, but when observed closely, localized enamel pitting could be seen on smooth surfaces of some premolars (Fig. 2B, 2C). No abnormality other than the thinner-than-normal layer of enamel was revealed on the radiograph (Fig. 2G). Her mother (II-1) reported the similar dental problems just like her daughter before her maxillary anterior teeth were covered with crowns.

#### Family 3

**Fig 3:**
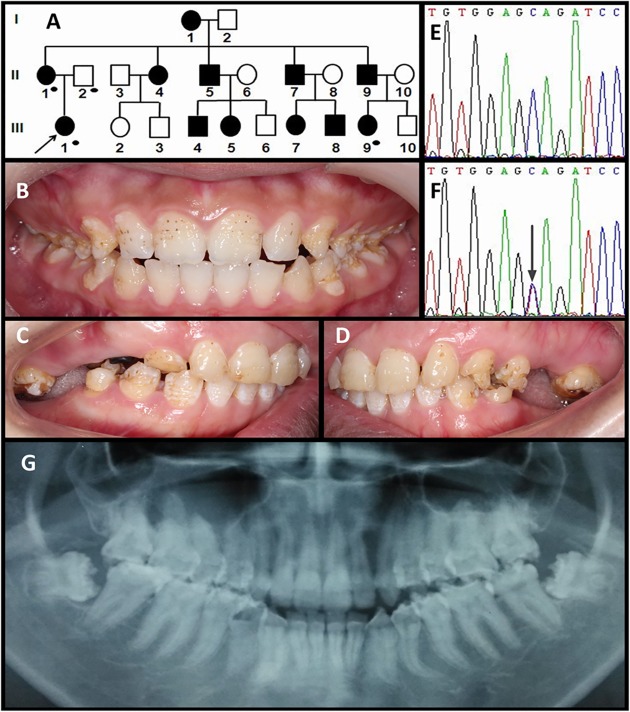
Clinical and mutation analysis of Family 3. (**A**) Pedigree of Family 3. Black dots indicate members recruited for this study. (**B**) Frontal clinical photograph of the 14.5-year-old proband. (**C, D**) Lateral clinical photographs of the proband's mother. (**E, F**) *LAMB3* exon 23 sequencing chromatogram of an unaffected family member (II:2) (E), and the proband (III:1) (F), revealed a nonsense mutation: c.3466C>T, p.Q1156X. (**G**) Panoramic radiograph of the proband.

In family 3, four family members were available (II-1, II-2, III-1 and III-9). The proband was a 14.5-year-old girl presenting with general hypoplasia in all permanent teeth. Her mother, who was the source of information concerning the pedigree and dental status of unexamined individuals, reported that affected family members had dental problems similar to those of the proband (Fig. 3A). The mother had lost most of her molars except for two upper teeth, with a total of 18 teeth still remaining (Fig. 3C and D). The girl displayed a distinctive pattern of enamel defects featuring deep irregular grooves and pits (Fig. 3B). No anterior open bite but a mild edge-to-edge occlusion was found in this proband. Her panoramic radiograph showed severe generalized enamel hypoplasia, but the enamel was generally more radiopaque than dentin. Excessive root canal calcification was evident in all erupted molars (Fig. 3G). No medical history of skin fragility or other syndromes was reported in this family.

### Mutation results

The *ENAM* mutation identified in Family 1 was a 19-bp insertion in exon 7 (c.406_407insTCAAAAAAGCCGACCACAA, p.K136Ifs*16) that shifted the reading frame and caused premature translation termination following the addition of 15 extraneous amino-acids. Analysis of her parents and grandparents revealed that the mutant allele came from her paternal lineage.

The AI-causing mutation in Family 2 was a single-base deletion in exon 5 of *ENAM* (c. 139delA, p. M47Cfs*11) also leading to the frameshift and a premature termination codon.

The proband of Family 3 had a *LAMB3* nonsense mutation in the last exon (c.3466C>T, p.Q1156X) that generated a premature termination codon with the absence of 17 C-terminal amino-acids. Sequence analysis of other participants revealed that the mutant allele came from her mother and perfectly segregated with the AI phenotype in this family. Besides, we also identified a missense mutation in exon 13 (c.1579G>A; p.V527M) in the proband's father (in heterozygous form) as well as in the AI patients (in homozygous form). However, with the SIFT algorithm [22,23], the amino acid substitution of V to M was predicted to be neutral to the function of the LAMB3 protein.

All these sequence variations identified in this study are not listed among the known polymorphisms in the current single-nucleotide polymorphism database (dbSNP, National Center for Biotechnology Information).

### Protein structure analysis

**Fig 4:**
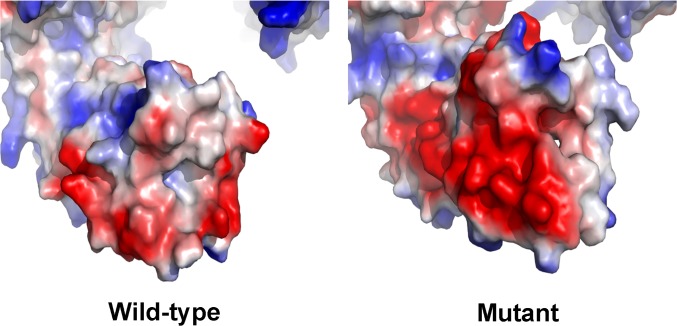
The electron-density distribution of the predicted C-terminus of the wild-type and mutant LAMB3 protein. The predicted C-terminal end are shown by electrostatic potential surfaces, which are differed extensively between the wild-type and mutant LAMB3 protein (blue represents positive potential; red, negative; white, neutral).

**Fig 5:**
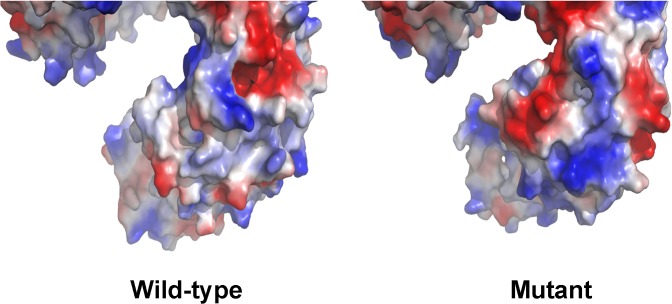
The electron-density distribution of C-terminus of the wild-type and mutant LAMB3 protein after 180° rotation. After 180° rotation, the difference of the electron-density distribution between the wild-type and mutant LAMB3 protein is shown.

After modeling the 3D structures of the wild-type and mutant LAMB3 proteins (c.3466C>T, p.Q1156X & c.1579G>A; p.V527M), we found the electron-density distribution (electrostatic surface potential) altered between the full-length and truncated LAMB3 proteins at the C-terminus (Figs. 4 and 5). The area of negative potential shown on mutant LAMB3 model was markedly enlarged in comparison to the wide-type one (Fig. 4). After 180e one (potthe distribution of three potentials differed from the wild-type as well (Fig. 5). We also performed protein structure analysis on the missense mutation (c.1579G>A; p.V527M) and the nonsense mutation (c.3466C>T, p.Q1156X), respectively. The missense mutation brought about very little change to the 3D protein structure, and the structure with only the nonsense mutation was almost the same as the one we modeled before (Figs. 4 and 5).

Therefore, based on the results of the SIFT prediction and the 3D protein structure models, we concluded that this missense mutation most probably was not involved in the etiology of hypoplastic AI in family 3.

## Discussion

In the present study, we found three novel heterozygous *ENAM* and *LAMB3* mutations (*ENAM*: c.406_407insTCAAAAAAGCCGACCACAA, p.K136Ifs*16; c. 139delA,p. M47Cfs*11, *LAMB3*: c.3466C>T, p.Q1156X) in three Chinese families with hypoplastic autosomal-dominant AI. Although AI types of these three families fell into the same category, the characteristics of their malformed enamel were different. In Family 1 and 2, the affected members mainly showed regular localized enamel hypoplasia, whereas those in Family 3 presented generalized, irregular pitting and grooves with molar crowns severely deformed. At present, up to 17 different *ENAM* mutations involving 6 exons have been reported, including missense, nonsense, frameshift and indel types [24,25]. The severity of the enamel defects sometimes varies widely, even among individuals with the same mutation [24,25]. The patterns of enamel defects we observed in Family 1 and 2 were similar to a Caucasian family, also with a frameshift *ENAM* mutation located within the fourth exon (c.107delA) [24]. By contrast, all previously reported mutations in the *LAMB3* gene prematurely terminated translation in the last two coding exons. The absence or substitution of the normal C-terminal amino-acids from the LAMB3 protein is sufficient to cause severe hypoplastic AI [19].

Amelogenesis is a highly-orchestrated process that not only involves enamel matrix proteins and their proteinases, but also depends on many other intracellular and membrane-associated proteins [8,12,26]. Prior to the secretory stage of enamel formation, the inner enamel epithelium rests on a basal lamina containing type-IV collagen, laminin-111, and laminin-332 [27]. After formation of the pre-dentin matrix, each pre-ameloblast elongates and shifts in polarity to become a polarized, secretory ameloblast. After they penetrate and remove the basal lamina, secretory ameloblasts begin to secrete proteins [15]. Thus, coordinated replacement of the basal lamina and pre-ameloblast polarization is presumably important for the initiation of enamel formation [19]. It has been proposed that laminin-332, together with integrin α3β1 plays a central role in the polarization and migration of cells [28,29]. Meanwhile, the developmental and tissue specificity of the expression of laminin-332 in the basal lamina during amelogenesis may also suggest its indispensable function in regulating ameloblast physiology. But unlike enamelin, the specific role of laminin-332 in enamel formation is unclear.

Laminin-332 is a basement-membrane protein composed of three constituent polypeptide chains, α3, β3, and γ2, encoded by the *LAMA3*, *LAMB3*, and *LAMC2* genes. Mutations in both alleles of *LAMA3*, *LAMB3*, and *LAMC2* can cause junctional epidermolysis bullosa (JEB) [30–32]. Among these, *LAMB3* mutations account for over half of all JEB cases [17]. When the function of LAMB3 protein is severely affected, individuals are likely to have JEB, whereas when this function is mildly affected, individuals are prone to have non-syndromic AI [19]. The novel mutation we report here brings the total number of non-syndromic AI mutations in the *LAMB3* gene to six. Since the premature translation termination codons in the last exon probably allow the transcripts to escape nonsense-mediated decay, the truncated LAMB3 protein is probably translated, and the phenotype is likely to be caused by the dominant-negative effects of expressing truncated LAMB3 that might function abnormally.

The three chains of laminin-332 share a common domain structure that consists of a short arm (globular and rod-like motifs) and a long arm (α-helical coiled-coil domain) [33]. The coiled-coil region is the only one connecting the three chains. At the N-terminal end of the long arm, all three chains are linked by disulfide bonds, while at the C-terminal end, only the β3- and γ2-chains are connected by a disulfide bond [34]. Previous work suggested that C-terminal trimerization of the α with β and γ chains is essential for the laminin globular (LG) domain (the C-terminal end of the α-chain containing five repeating LG domains) to exert its integrin-binding activity [35]. And integrin-mediated interactions with the C-terminus of laminins are crucial for several cellular activities by activating specific signaling networks governing adhesion, migration, and differentiation [36]. Among the laminin-binding integrins, α3β1 and α6β4 bind exclusively to laminins containing the α3 chain [37]. A lack of integrin α3β1-binding activity in the absence of short β3 segment has been shown for α3 LG1-LG3 [38,39]. This also suggests that the C-terminal region of laminin β-chains help modulate the integrin-binding affinities of laminins [40]. In addition, electrostatic forces and energies are essential for the interactions between macromolecules [41], and some protein-protein interactions need to be electrostatically guided [42,43]. Based on our predicted models, the deletion of 17 amino-acids from the C-terminal region likely alter local electron-density distribution of mutant LAMB3 protein, and this may contribute to disturbance of the binding activity of the heterotrimer.

In conclusion, heterozygous mutations in the *ENAM* and *LAMB3* genes can cause hypoplastic AI with markedly different phenotypes in Chinese patients. Our results extend the mutation spectrum of both genes and may be used for mutation screening of AI in the Chinese population. However, the underlying mechanism by which LAMB3 protein is involved in enamel formation remains to be discovered.

## Supporting Information

S1 Table Primers for amplifying *ENAM* (exon 1–10). (DOCX)

S2 Table Primers for amplifying *LAMB3* (exon 1–23). (DOCX)

S1 Fig The predicted 3D protein model of the C-terminus of LAMB3 protein. Cyan indicates the wild-type; Green indicates the mutant; Red indicates the absent part of the truncated protein.
(DOCX)
