# Supplementary material for: Novel ENAM and LAMB3 Mutations in Chinese Families with Hypoplastic Amelogenesis Imperfecta
Source: PLoS One. 2015 Mar 13;10(3):e0116514. doi: 10.1371/journal.pone.0116514 (PMC4358960; doi:10.1371/journal.pone.0116514)
Supplement: S1 Table — (DOCX) [file pone.0116514.s001.docx]

**Table S1: Primers for amplifying *ENAM* (exon 1-10)**

| gagacttgacttgacagctcctat | ENAM 1-F |  |
| --- | --- | --- |
| tctctaatactcacccaatgcc | ENAM 1-R |  |
| tccagccttcttagacctccttt | ENAM 2-F |  |
| ttctaaggcagcacatgtcatt | ENAM 2-R |  |
| tgctgccttagaactgaagc | ENAM 3-F |  |
| tgagaacctgtatgtattggc | ENAM 3-R |  |
| ccatactctccttgacagacaa | ENAM 4,5-F |  |
| tactttgcctcgatttgagagt | ENAM 4,5-R |  |
| cactgggaagttctaaggtt | ENAM 6-F |  |
| aacggagttatctagataaacaag | ENAM 6-R |  |
| cagcctgaatcacagctctatt | ENAM 7-F |  |
| ttaaaaggcaacagtatttgggta | ENAM 7-R |  |
| ttatcattatcgtctttgccctat | ENAM 8-F |  |
| cccagtttccccattacatt | ENAM 8-R |  |
| tcgaacgtggttttctcctgtgtt | ENAM 9-F |  |
| agcaggggcgaatggattgt | ENAM 9-R |  |
| tccaaacaacaccatggtgg | ENAM 10A-F |  |
| ccctttaggatttggttggg | ENAM 10A-R |  |
| agaaagtccaggcacagaac | ENAM 10B-F |  |
| atttggctgacttggtctcc | ENAM 10B-R |  |
| ctgcagtcaacgcttcagg | ENAM 10C-F |  |
| actggatttcctggacgagc | ENAM 10C-R |  |
| cctcggtggaacttctttgc | ENAM 10D-F |  |
| ttgttcttttggacccaggg | ENAM 10D-R |  |
| tggccctgttgttcgcaatg | ENAM 10E-F |  |
| ggtttgtccatcagaatttgg | ENAM 10E-R |  |
| aaactcctattacccaagagg | ENAM 10F-F |  |
| gagagatttcttggtggtcc | ENAM 10F-R |  |
| gaggaaatcccttctcctgc | ENAM 10G-F |  |
| cccatagtattgggagaatc | ENAM 10G-R |  |
| ccttagaggcaatacatggg | ENAM 10H-F |  |
| ttcctttggctgatttgagg | ENAM 10H-R |  |
| acagttaggcactatgaagg | ENAM 10J-F |  |
| tcccaggaattccgtgaagg | ENAM 10J-R |  |
| gttaataatgccgctggacc | ENAM 10K-F |  |
| gccatagttcaaattctcacc | ENAM 10K-R |  |
| gccctattacagtaacaccc | ENAM 10L-F |  |
| gtagagctagtggattgtcc | ENAM 10L-R |  |
| tcttgctgtgctggtagctc | ENAM 10M-F |  |
| agaacatggtgtgttcctcc | ENAM 10M-R |  |
| ccttttagagatgatgtgtcc | ENAM 10N-F |  |
| ggattggagccttcatcagg | ENAM 10N-R |  |
| tgaccttactcctgagcagc | ENAM 10P-F |  |
| cctcagtagctaactcaacc | ENAM 10P-R |  |
| gggattcaattacgcctactg | ENAM 10Q-F |  |
| cagacacttaattaagtcttgg | ENAM 10Q-R |  |
