# Supplementary material for: Novel ENAM and LAMB3 Mutations in Chinese Families with Hypoplastic Amelogenesis Imperfecta
Source: PLoS One. 2015 Mar 13;10(3):e0116514. doi: 10.1371/journal.pone.0116514 (PMC4358960; doi:10.1371/journal.pone.0116514)
Supplement: S2 Table — (DOCX) [file pone.0116514.s002.docx]

**Table S2: Primers for amplifying *LAMB3* (exon 1-23)**

| agtgacgacagatgggtgtg | LAMB-1F |
| --- | --- |
| ccggatctgaacctgctttc | LAMB-1R |
| gctgctccaagtgtcctaga | LAMB-2F |
| tgcctccctgccatataaca | LAMB-2R |
| agtggaagcttgagtgaggg | LAMB-3F |
| ttcagtggacaaatggcagc | LAMB-3R |
| gctatgtgttaggaggggca | LAMB-4F |
| tgagttcccgtagatggcaa | LAMB-4R |
| ctggggaagtggacagtctc | LAMB-5F |
| agcgagtcctcttctgtcac | LAMB-5R |
| ccctttctcccgagttcact | LAMB-6F |
| cccaggcctacttttcagga | LAMB-6R |
| tgaactcggggcacactatt | LAMB-7F |
| ccagacctcaaccatcccat | LAMB-7R |
| cttccctccttgctgtttgg | LAMB-8F |
| atgtctgtagtctgccctgg | LAMB-8R |
| cctggtgcccttctttgttg | LAMB-9F |
| cagagccaggagagcttgaa | LAMB-9R |
| aggtgtgggtcagatgtcag | LAMB-10F |
| tgtgcccaggaacatgtact | LAMB-10R |
| ctttgttcctactgcggtgg | LAMB-11F |
| tgaagagagcacagtgagca | LAMB-11R |
| ccagttcttttcccaggcag | LAMB-12F |
| tgagaacggggacagaagac | LAMB-12R |
| agccctggattctacctgtg | LAMB-13F |
| caccacatgcatcagtacagg | LAMB-13R |
| tctggctgcgacttctgtta | LAMB-14F |
| ctggaacccctggagcataa | LAMB-14R |
| ttttgtggctccatctgcac | LAMB-15F |
| aatggagatggaggaggagc | LAMB-15R |
| gctcctcctccatctccatt | LAMB-16F |
| gttctcacaggggcagatct | LAMB-16R |
| ggtgcttggttgtcatggag | LAMB-17F |
| atcctgtctgcctcctccta | LAMB-17R |
| ccacatgtaagctgggttgg | LAMB-18F |
| tgcactgaacatgggaatgc | LAMB-18R |
| cttccacaagtgctgctcag | LAMB-19F |
| tgggcagagagaagttcagg | LAMB-19R |
| ggtgtcttgggcataatggg | LAMB-20F |
| caaatatgggcggaggaagc | LAMB-20R |
| ttttgctgcctgatgagttg | LAMB 21F |
| tggagggagaaatcagatgc | LAMB 21R |
| cattgtcctgggagtgaggt | LAMB 22F |
| gcacggctagctccaataag | LAMB 22R |
| tttttctggaggggattcct | LAMB 23F |
| cccagcttccttgacttgag | LAMB 23R |
