# Supplementary material for: Novel ENAM and LAMB3 Mutations in Chinese Families with Hypoplastic Amelogenesis Imperfecta
Source: PLoS One. 2015 Mar 13;10(3):e0116514. doi: 10.1371/journal.pone.0116514 (PMC4358960; doi:10.1371/journal.pone.0116514)

**Figure S1:** **The predicted 3D protein model of the C-terminus of LAMB3 protein.** Cyan indicates the wild-type; Green indicates the mutant; Red indicates the absent part of the truncated protein.


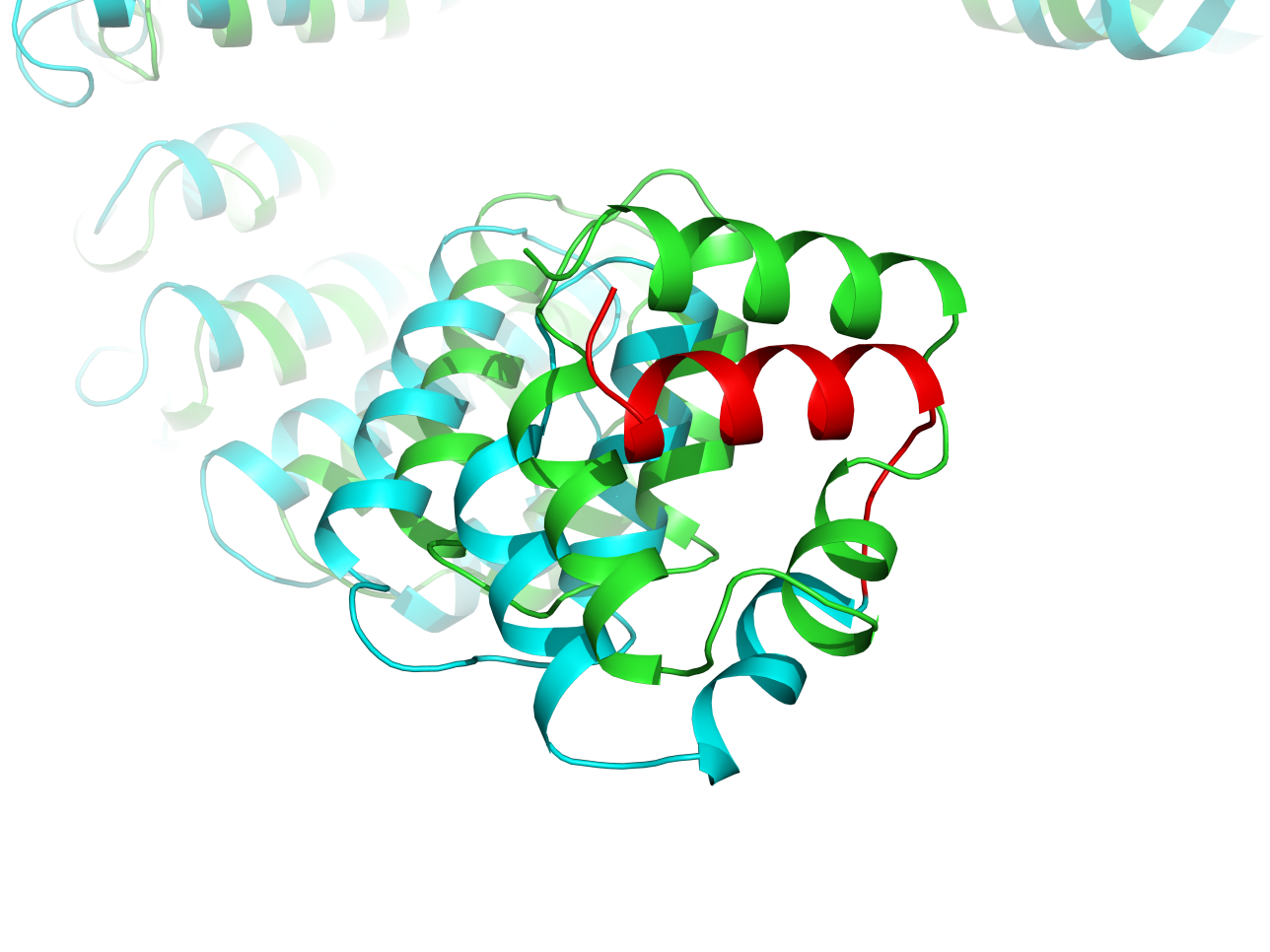

Supplement: S1 Fig — Cyan indicates the wild-type; Green indicates the mutant; Red indicates the absent part of the truncated protein. (DOCX) [file pone.0116514.s003.docx]
